# The effect of depressive symptoms on quality of life and its different facets in the oldest age population: evidence from the AgeQualiDe prospective cohort study

**DOI:** 10.1007/s11136-023-03526-7

**Published:** 2023-10-28

**Authors:** Paula Liegert, Alexander Pabst, Ines Conrad, Hendrik van den Bussche, Marion Eisele, André Hajek, Kathrin Heser, Luca Kleineidam, Siegfried Weyerer, Jochen Werle, Michael Pentzek, Dagmar Weeg, Edelgard Mösch, Birgitt Wiese, Anke Oey, Michael Wagner, Wolfgang Maier, Hans-Helmut König, Steffi G. Riedel-Heller, Martin Scherer, Melanie Luppa

**Affiliations:** 1 Medical Faculty, University of Leipzig, Leipzig, Germany; 2 Institute of Social Medicine, Occupational Health and Public Health, University of Leipzig, Leipzig, Germany; 3 Department of Primary Medical Care, Center for Psychosocial Medicine, University Medical Center Hamburg-Eppendorf, Hamburg, Germany; 4 Department of Health Economics and Health Services Research, Hamburg Center for Health Economics, University Medical Center Hamburg-Eppendorf, Hamburg, Germany; 5 Department of Neurodegenerative Diseases and Geriatric Psychiatry, University Hospital Bonn, Bonn, Germany; 6 German Center for Neurodegenerative Diseases (DZNE), Bonn, Germany; 7 Central Institute of Mental Health, Medical Faculty Mannheim, Mannheim, Germany; 8 Institute of General Practice, Medical Faculty, Heinrich-Heine-University Düsseldorf, Düsseldorf, Germany; 9 Department of Psychiatry, Technical University of Munich, Munich, Germany; 10 Institute of General Practice, Hannover Medical School, Hannover, Germany

**Keywords:** Quality of life, QoL, Depressive symptoms, Depression, Oldest age, *WHOQOL-OLD*, *GDS-15*, Longitudinal study

## Abstract

**Purpose:**

The present study aims to investigate the prospective effect of depressive symptoms on overall QoL in the oldest age group, taking into account its different facets.

**Methods:**

Data were derived from the multicenter prospective *AgeCoDe/AgeQualiDe* cohort study, including data from follow-up 7–9 and *n* = 580 individuals 85 years of age and older. Overall QoL and its facets were assessed using the *WHOQOL-OLD* instrument. The short form of the *geriatric depression scale* (*GDS-15*) was applied to assess depressive symptoms. Cognitively impaired individuals were excluded. Linear mixed-effects models were used to assess the effect of depressive symptoms on QoL.

**Results:**

Depressive symptoms were significantly associated with overall QoL and each of the different facets of WHOQOL-OLD, also after adjustment for time and sociodemographic characteristics such as age, gender, education, marital status, living situation, and cognitive status. Higher age and single as well as divorced marital status were also associated with a lower QoL.

**Conclusion:**

This work provides comprehensive longitudinal results on the relationship between depressive symptoms and QoL in the oldest age population. The results underscore the relevance of tailored and targeted care planning and the development of customized interventions.

## Introduction/background

The demographic change leads to an increasing proportion of oldest people around the world. Approximately every eights person will be 80 years old and older in Germany in 2060 [1]. Depression is known to be one of the most common mental disorders in old age, with an increasing incidence in the oldest age group [2, 3]. A meta-analysis of population-based studies showed a cumulative prevalence of dimensionally measured depressive symptoms of 17% [4]. Especially in the oldest age population numerous life changes occur such as loss or disability of close relatives or friends, physical illness or impairment, or wide-ranging changes of financial or other life circumstances that are often associated with the development of depressive symptoms [e.g., 5, 6], and a decrease in quality of life (QoL) [7].

According to the World Health Organization Quality of Life Group [8, 9] Quality of life (QoL) is defined as “an individual’s perception of their position in life in the context of the culture and value system in which they live and in relation to their goals, expectations, standards, and concerns”. QoL can be captured in general terms and/or in its different facets, which may include physical, psychological, social, environmental, and spiritual aspects. The WHOQOL-OLD—which was used in the present study—is a multidimensional instrument comprising a total QoL score and six QoL facets especially adapted to the elderly population: *sensory abilities*, *autonomy*, *past, present and future activities*, *social participation*, *death and dying*, and *intimacy* [10].

Until now, the association of depressive symptoms and QoL has been frequently investigated in the elderly population. Most studies reported a strong association of depressive symptoms and a lower overall QoL, however, most of them used a cross-sectional study design as shown by the systematic review of Sivertsen et al. [7]. Of the 74 included studies, 52 had a cross-sectional design; of the 22 studies using a longitudinal design, only 10 were community-based and only one study reported results for the oldest age population [11], but were restricted to overall QoL.

In sum, less is known about the effect of depressive symptoms on overall QoL in the oldest population from a longitudinal perspective, even less about the effect of depressive symptoms on different facets of QoL in the general elderly population [7], and—to the best of our knowledge—nothing about the effect of depressive symptoms on overall QoL and its facets in the oldest age group. However, especially the investigation of the different facets of QoL is of high relevance in the oldest population since these individuals experience a lot of alteration in functional, social and mental dimensions, such as vision and hearing functioning, in the relationship with loved ones, experience loneliness and loss of personal autonomy, or change of attitudes toward death and dying [10, 12].

Thus, the present study aims to investigate the prospective effect of depressive symptoms on QoL in the oldest population, focusing on overall QoL and its different facets using data from a large German multicentered, prospective primary care study of the aged population.

## Methods

### Study design and sample

Data from the 'Study on needs, health service use, costs, and health-related quality of life in a large sample of oldest-old primary care patients (85 +)' (AgeQualiDe) were used in the present study. This multicenter prospective cohort study (cites: Bonn, Düsseldorf, Hamburg, Leipzig, Mannheim, Munich) is the continuation (from follow-up 7 to follow-up 9) and extension with regard to the content of the “German Study on Ageing, Cognition, and Dementia in Primary Care Patients” (AgeCoDe; from baseline to follow-up 6).

The baseline assessment of the AgeCoDe study was carried out in 2003/2004; a total of *n* = 3327 eligible GP patients consented to participate. Inclusion criteria at baseline of the AgeCoDe study were: >  = 75 years, absence of dementia, and at least one contact with a GP within the preceding 12 months. Exclusion criteria were as follows: GP consultations through home visits only, nursing home residence, severe illness with expected fatal outcome within 3 months, German language insufficiency, deafness or blindness, and inability to provide informed consent. Individuals and their proxies were interviewed by trained physicians and psychologists. Further methodical details have been described in detail elsewhere [13, 14].

**Fig. 1:**
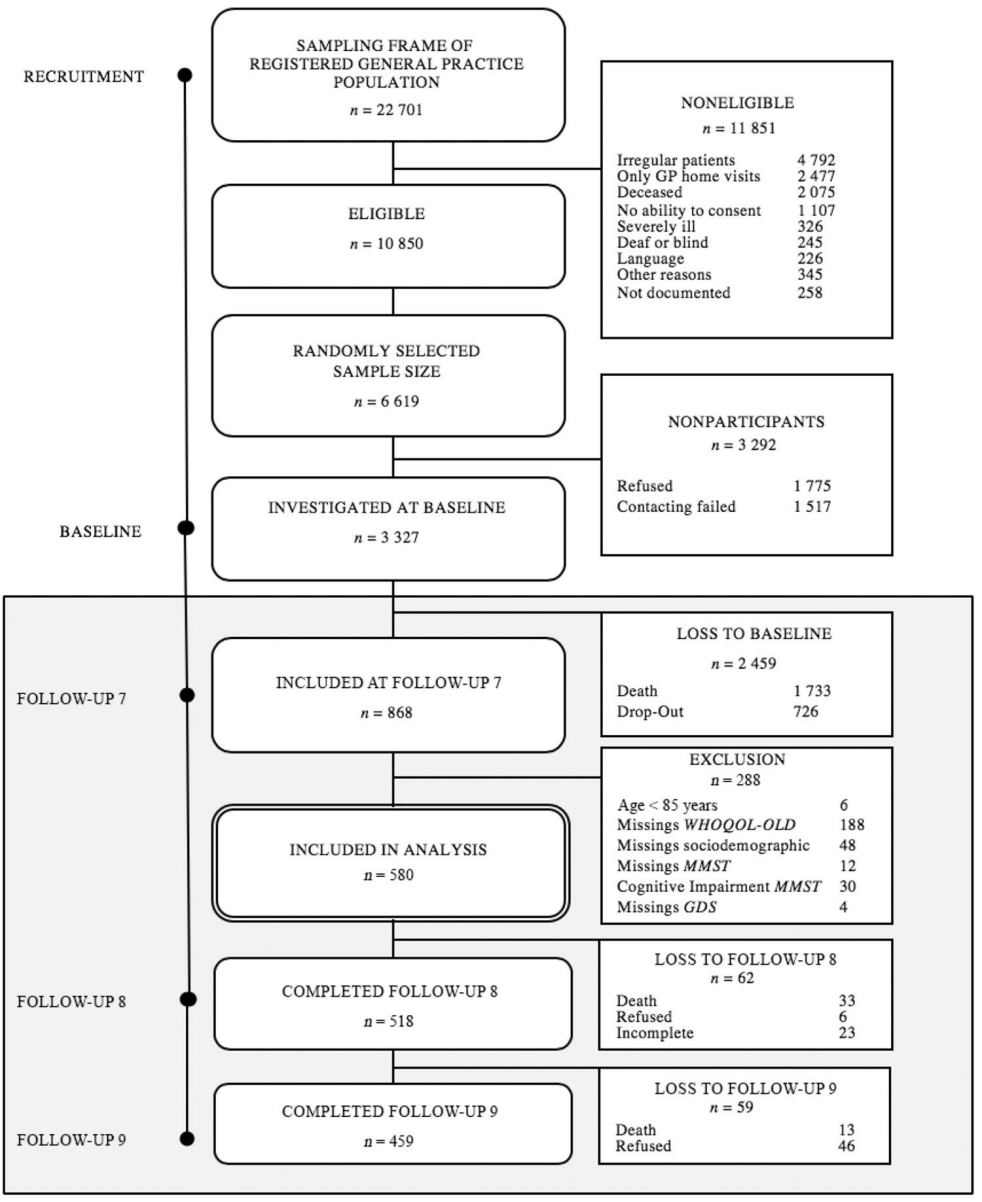
Flowchart of the sample selection process

The present study used data from the follow-ups 7–9 for reasons of data availability, especially regarding quality of life. At follow-up 7 (2014/15), the sample consisted of *n* = 868 patients, meanwhile aged of 85 years and older. Of these, 288 participants had to be excluded: 30 participants had a Mini Mental State Examination (MMSE) score less than 24 points, therefore showing significant cognitive impairment limiting validity of self-report of depressive symptoms and QoL; 252 persons were excluded due to incomplete, invalid, or missing values in the relevant variables; another 6 persons were under an age of 85 years. Finally, 580 individuals were included in the study sample (66.8%), and further examined in the follow-ups 8 and 9 every 10 months until 2016. The excluded participants (*n* = 288) were somewhat older (89.6 vs. 88.6 years; *p* < 0.001) and more often male (34.5 vs. 24.3%; *p* < 0.01) than participants with complete information included in the analytic sample, and did not differ in terms of education. Figure 1 shows a flowchart of the sample selection process, including detailed information on sampling attrition of the data used in the present study (follow-up 7–9).

Of the 580 participants, 62 (10.2%) were lost to FU8, and in total 121 (20.9%) were lost to FU9. Drop outs to FU9 were more often male, had a lower MMSE score, a higher GDS score and lower WHOQOL-OLD overall score as well as lower scores in each QoL facet, except for activities, and for death and dying (see Appendix A).

### Data collection and assessment procedures

Structured clinical interviews were conducted collecting sociodemographic, neuropsychological, and health data from the participants.

### Dependent variable

Quality of life (QoL) was assessed using the World Health Organization Quality of Life Assessment for Older Adults (WHOQOL-OLD) [12, 15, 16]. The WHOQOL-OLD consists of 24 items developed to assess the specificities of QoL in older adults using an ordinal 5-point-Likert scale for assessment. It is composed of six facets, *sensory abilities*, *autonomy*, *past, present and future activities*, *social participation*, *death and dying*, and *intimacy* and provides a total QoL score with a higher score indicating a better QoL.

In the AgeQualiDe study, five of the six facets were assessed, since—by the principle investigators of the study—the facet intimacy was considered not reasonable for the very old predominantly widowed study participants in order to avoid drop outs and missing data. The WHOQOL-OLD shows good psychometric characteristics [12, 15]. QoL overall scores and facets were calculated for each of the three follow-up waves 7–9.

### Independent variables

Data from the following predictor variables, assessed at follow-up 7, were considered: First, depressive symptoms were identified using the short version of the geriatric depression scale (GDS-15) [17]. The GDS is a self-rated depression screening instrument that is implemented in a wide range of geriatric care and science settings [18]. The German short form (GDS-15) consists of 15 items with a sum score ranging from 0 to 15 [19]. It has a simplified yes/no-response format and excludes questions for somatic symptoms. For that reason, it is especially suitable for the older and oldest old population. A cut-off point of 5 indicates clinically relevant depressive symptoms [18, 20, 21].

Second, the cognitive status of the participants was assessed using the MMSE (total score range 0–30; a higher score indicates higher cognitive function) [22]. Participants with a MMSE score lower than 24 were excluded from the analyses, because of the limited validity of self-report of depressive symptoms in the GDS and related to QoL [22]. Cognitive status was used as covariate in the regression models.

Finally, sociodemographic characteristics including age, gender, marital status, and living situation were considered to account for potential confounding. The educational level was classified as low, medium or high according to the new Comparative Analysis of Social Mobility in Industrial Nations (CASMIN) educational classification [23].

### Statistical analysis

Data were entered into centers through an Internet-based remote data entry system into a central ORACLE database, version 9. Statistical analyzes were performed using the SPSS version 27 statistical software for Microsoft Windows and Stata SE version 16.1 (StataCorp LP, College Station, TX). The level of statistical significance was established at *p* < 0.05 (two-tailed) for all analyzes.

Descriptive data are presented as means with standard deviations or frequencies with percentages, as appropriate.

The differences in baseline characteristics (follow-up 7) between subjects with and without depressive symptoms were analyzed using *χ*2 test for categorical variables or *t-*tests for continuous variables. To investigate the effect of depressive symptoms on QoL, we used multilevel mixed-effects linear regression models, since all outcomes approximate a normal distribution. These models allow controlling for unobserved individual heterogeneity in panel data. Thus, missing information over time is handled autonomously so that unbalanced data do not result in the exclusion of cases. All models included a fixed effect for time (reference follow-up 7) to control for overall temporal variation in QoL (facets), a random intercept to allow for heterogeneity across participants, and an autoregressive covariance structure to account for autocorrelation due to the temporal dependencies of repeated measurements over time. The clustered design of the study was accounted for by including a clustered sandwich estimator for GP to correct the standard errors of model estimates.

First, unadjusted linear mixed-effects models were performed for the association of depressive symptoms with QoL (total score and facets) averaged across all follow-ups. Second, adjusted linear mixed-effects models were performed for the association of depressive symptoms with QoL scores adjusted for potential confounders, including age (continuous), gender (male vs. female), education (low, middle vs. high), living situation (private home vs. institutionalized living), marital status (single, married/cohabiting, divorced vs. widowed) and cognitive functioning (MMSE, continuous). Results are shown as effect estimators (betas) with corresponding confidence intervals and standard errors. Wald test are reported to indicate significance of categorical predictor variables.

## Results

### Sample characteristics

Table 1 summarizes the sample characteristics of the total sample (n = 580) and of the participants with and without depressive symptoms. Among the 580 participants in the study sample, 100 (17.2%) had a GDS-15 cutoff score of < 5 indicating depressive symptoms at baseline (mean (standard deviation (SD) of the GDS: 7.1 (1.6) vs. 2.1 (1.3)*, p* < 0.001).

**Table 1:** Socio-demographic and health characteristics of the analytical sample of GP patients at FU7 (*n* = 580)

Variables	Total (*n* = 580)	Depressive symptoms *GDS-15* ≥ 5 (*n* = 100; 17.2%)	No depressive symptoms *GDS-15* ≤ 4 (*n* = 480; 82.8%)	*p-*value
Age				
Range	85–99			
* M (SD)*	88.6 (2.7)	88.8 (2.7)	88.6 (2.7)	0.352
Sex (*n*/%)				
Female	380 (65.5)	74 (74.0)	306 (63.8)	0.057
Education (*n*/%)^a^				
Low	303 (52.2)	52 (52.0)	251 (52.3)	0.406
Middle	179 (30.9)	35 (35.0)	144 (30.0)	
High	98 (16.9)	13 (13.0)	85 (17.7)	
Marital status (*n*/%)				
Single	36 (6.2)	10 (10.0)	26 (5.4)	< 0.01
Married	157 (27.1)	15 (15.0)	142 (29.6)	
Divorced	28 (4.8)	11 (11.0)	17 (3.5)	
Widowed	359 (61.9)	64 (64.0)	295 (61.5)	
Living situation (n/%)				0.533
private home	536 (92.4)	91 (91.0)	445 (92.7)	
institution	44 (7.6)	9 (9.0)	35 (7.3)	
*MMSE*^b^				
Range	24–30			
* M (SD)*	28.0 (1.6)	27.9 (1.6)	28.1 (1.6)	.351
*GDS-15*^c^				
Range	0–14			
* M (SD)*	2.6 (2.5)	7.1 (2.1)	1.6 (1.3)	< 0.001
*WHOQOL-OLD*^d^				
Range	19–80			
* M (SD)*	55.3 (9.6)	46.2 (8.8)	57.1 (8.7)	< 0.001
* Sensory abilities*	55.6 (17.1)	47.8 (18.8)	57.2 (16.3)	< 0.001
* Autonomy*	53.1 (13.8)	44.3 (13.4)	55.0 (13.1)	< 0.001
* Activities*	55.0 (10.9)	45.4 (10.9)	57.0 (9.9)	< 0.001
* Social participation*	53.3 (13.7)	39.3 (13.3)	56.2 (11.9)	< 0.001
* Death and dying*	59.4 (18.2)	54.4(20.0)	60.4 (17.7)	< 0.01

*n* frequencies, % percent, *M* Mean; *SD* Standard deviation

^a^*a*ccording to the new CASMIN educational classification. Low = inadequately completed general education, general elementary education, basic vocational qualification or general elementary education and vocational qualification; Middle = intermediate vocational qualification or intermediate general qualification and vocational qualification, intermediate general qualification, general maturity certificate, vocational maturity certificate/general maturity certificate and vocational qualification; High = lower tertiary education-general diplomas/diplomas with vocational emphasis, higher tertiary education-lower level/higher level [23]

^b^*MMSE* = *Mini-Mental-Status-Examination* [22]; ^c^*GDS-15* = *Geriatric Depression Scale* (short-form) [17–19]

^d^*WHOQOL-OLD* = *World Health Organization Quality of Life-OLD*-questionnaire [9, 10]

The age ranged from 85 to 99 years, with a mean age of 88.6 years (*SD* = 2.7). Two-thirds (66%) of the sample was female. Half of the sample had a low educational level (52.2%). The majority (92.4%) of the participants lived in a private home. Almost two thirds (61.9%) were widowed and a large proportion was married (27.1%). Persons with relevant depressive symptoms were more likely to be single, widowed, or divorced than persons without relevant depressive symptoms.

### Association of depressive symptoms and quality of life

A comparison of both groups of people with and without depressive symptoms showed that the presence of significant depressive symptoms determined significantly lower levels of QoL (mean/SD: 46.2/8.8 vs. 57.1/8.7, *p* < 0.001). This association was also found for all WHOQOL-OLD facets (see Table 1).

In Table 2, the results of the unadjusted and adjusted linear mixed-effects models of significant depressive symptoms are shown on the WHOQOL-OLD total score. In both models, a significant effect of depressive symptoms on the WHOQOL-OLD scores was shown, also after adjustment for the relevant covariates. Higher age and single as well as divorced marital status were also associated with a lower overall QoL score.

**Table 2:** Results of the unadjusted and adjusted linear mixed effects regression models: depressive symptoms and WHOQOL-OLD total score over the course of the study period (*n* = 580)

	Effect estimates (95% ci)	Standard error	*p*-value
QOL-Total			
Unadjusted model			
Time (ref. FU7)	χ^2^ = 13.88 (*p* < 0.01)		
FU8	− 0.43 (− 1.02; 0.16)	0.30	0.149
FU9	− 1.30 (− 2.00; − 0.61)	0.35	< 0.001
Depressive symptoms (*GDS-15*^b^)	− 2.10 (− 2.36, − 1.85)	0.13	< 0.001
Adjusted model			
Time (ref. FU7)	χ^2^ = 13.41 (*p* < 0.01)		
FU8	− 0.43 (− 1.02; 0.16)	0.30	0.152
FU9	− 1.29 (− 1.98; − 0.59)	0.36	< 0.001
Depressive symptoms (*GDS-15*^b^)	− 2.00 (− 2.25, − 1.74)	0.13	< 0.001
Age, every additional year	− 0.36 (− 0.62, − 0.09)	0.14	0.008
Gender, male	1.17 (− 0.22, 2.56)	0.71	0.100
Education^c^ (ref. high)	χ^2^ = 0.41 (*p* = 0.813)		
Low	0.56 (− 1.27, 2.39)	0.93	0.550
Middle	0.35 (− 1.75, 2.44)	1.07	0.745
Living situation, (ref. private home)			
Institution	1.75 (− 0.03, 3.52)	0.91	0.054
Marital status (ref. widowed)	χ^2^ = 9.21 (*p* < 0.05)		
Single	− 3.02 (− 5.55, − 0.50)	1.29	0.019
Married	− 0.49 (− 2.12, 1.15)	0.83	0.560
Divorced	− 2.94 (− 5.40, − 0.47)	1.26	0.020
Cognitive functioning (*MMSE*^d^)	− 0.24 (− 0.63, 0.14)	0.20	0.217

95% CI = Confidence interval

^a^*WHOQOL*-*OLD* = *World Health Organization Quality of Life-OLD*-questionnaire [9, 10]

^b^*GDS-15* = *Geriatric Depression Scale* (short-form) [17–19]

^C^*a*ccording to the new CASMIN educational classification. Low = inadequately completed general education, general elementary education, basic vocational qualification or general elementary education and vocational qualification; Middle = intermediate vocational qualification or intermediate general qualification and vocational qualification, intermediate general qualification, general maturity certificate, vocational maturity certificate/general maturity certificate and vocational qualification; High = lower tertiary education-general diplomas/diplomas with vocational emphasis, higher tertiary education-lower level/higher level [23]

^d^*MMSE* = *Mini-Mental-Status-Examination* [22]

In Table 3, the results of the unadjusted and adjusted linear mixed-effects models of significant depressive symptoms are displayed on the WHOQOL-OLD facets scores. In both models, a significant effect of depressive symptoms was shown on every WHOQOL-OLD facets score, also after adjustment for the relevant covariates. A higher age was also associated with a lower quality of life in the *sensory abilities* and *social participation*. Male gender was associated with a higher QoL in the facets *activities* as well as *death and dying*. Participants with a single marital status showed a lower quality of life in the facets *activities* and *social participation* compared to widowed participants. Divorced participants also showed a lower quality of life in facet *activities* compared to widowed participants. A higher cognitive status was associated with a higher quality of life in facet *autonomy* and with a lower quality of life in facet *death and dying.*

**Table 3:** Results of the unadjusted and adjusted linear mixed effects regression models: depressive symptoms and WHOQOL-OLD facets scores over the course of the study period (*n* = 580)

	Sensory abilities			Autonomy			Past, present, and future activities			Social participation			Death and dying		
	Effect estimates (95% CI)	Standard error	*p*-value	Effect estimates (95% CI)	Standard error	*p*-value	Effect estimates (95% CI)	Standard error	*p*-value	Effect estimates (95% CI)	Standard error	*p*-value	Effect estimates (95% CI)	Standard error	*p*-value
Unadjusted model															
Time (ref. FU7)	χ^2^ = 17.43 (*p* < 0.001)			χ^2^ = 15.08 (*p* < 0.001)			χ^2^ = 11.90 (*p* < 0.01)			χ^2^ = 32.68 (p < 0.001)			χ^2^ = 17.18 (*p* < 0.001)		
FU8	− 0.26 (− 1.31, 0.79)	0.54	.623	− 0.80 (− 1.99, 0.39)	0.61	.186	− 0.94 (− 1.71, − 0.16)	0.40	.018	− 2.26 (− 3.23, − 1.28)	0.50	< 0.001	2.24 (1.04, 3.43)	0.61	< 0.001
FU9	− 2.26 (− 3.52, − 1.00)	0.64	< .001	− 2.51 (− 3.78, − 1.23)	0.65	< 0.001	− 1.58 (− 2.52, − 0.64)	0.48	0.001	− 3.12 (− 4.32, − 1.93)	0.61	< 0.001	3.07 (1.32, 4.82)	0.89	0.001
Depressive symptoms (*GDS-15*^b^)	− 1.92 (− 2.53, − 1.32)	0.31	< 0.001	− 2.11 (− 2.52, − 1.70)	0.21	< 0.001	− 2.21 (− 2.49, − 1.94)	0.14	< 0.001	− 3.06 (− 3.36, − 2.76)	0.15	< 0.001	− 1.21 (− 1.69, − 0.73)	0.24	< 0.001
Adjusted model															
Time (ref. FU7)	χ^2^ = 17.65 (*p* < 0.001)			χ^2^ = 15.84 (*p* < 0.001)			χ^2^ = 11.56 (*p* < 0.01)			χ^2^ = 33.31 (*p* < 0.001)			χ^2^ = 18.57 (*p* < 0.001)		
FU8	− 0.27 (− 1.31, 0.78)	0.53	.617	− 0.83 (− 2.02, 0.36)	0.61	.170	− 0.93 (− 1.71, − 0.16)	0.40	0.019	− 2.28 (− 3.26, − 1.31)	0.50	< 0.001	2.29 (1.10, 3.48)	0.61	< 0.001
FU9	− 2.26 (− 3.50, − 1.02)	0.63	< 0.001	− 2.59 (− 3.88, − 1.30)	0.66	< 0.001	− 1.57 (− 2.52, − 0.63)	0.48	0.001	− 3.17 (− 4.36, − 1.98)	0.61	< 0.001	3.22 (1.47, 4.96)	0.89	< 0.001
Depressive symptoms (*GDS-15*^b^)	− 1.76 (− 2.36, − 1.16)	0.31	< 0.001	− 2.06 (− 2.47, − 1.64)	0.21	< 0.001	− 2.07 (− 2.35, − 1.79)	0.14	< 0.001	− 2.93 (− 3.24, − 2.62)	0.16	< 0.001	− 1.18 (− 1.66, − 0.70)	0.24	< 0.001
Age	− 1.48 (− 1.99, − 0.97)	0.26	< 0.001	− 0.24 (− 0.61, 0.14)	0.19	.218	− 0.10 (− 0.37, 0.17)	0.14	0.473	− 0.40 (− 0.76, − 0.03)	0.19	0.036	0.39 (− 0.07, 0.85)	0.23	0.094
Gender, male	0.19 (− 2.67, 3.04)	1.46	0.898	0.50 (− 1.97, 2.97)	1.26	0.691	1.81 (0.07, 3.54)	0.88	0.041	− 0.84 (− 2.87, 1.19)	1.04	0.420	4.41 (1.86, 6.96)	1.30	0.001
Education^c^ (ref. high)	χ^2^ = 1.07 (*p = 0.58*6)			χ^2^ = 0.61 (*p* = 0.735)			χ^2^ = 0.85 (*p* = 0.653)			χ^2^ = 1.55 (*p* = 0.461)			χ^2^ = 1.24 (*p* = 0.537)		
low	1.59 (− 2.19, 5.37)	1.93	.409	− 0.92 (− 3.33, 1.48)	1.23	.452	− 0.11 (− 2.14, 1.92)	1.04	.916	0.13 (− 2.11, 2.37)	1.14	0.910	1.81 (− 1.56, 5.17)	1.72	0.293
middle	2.07 (− 1.88, 6.01)	2.01	0.304	− 0.93 (− 3.56, 1.70)	1.34	0.487	− 0.69 (− 2.82, 1.44)	1.09	0.525	− 0.86 (− 3.45, 1.73)	1.32	0.516	1.83 (− 1.78, 5.45)	1.84	0.320
Living situation, (ref. private home) institution	3.24 (− 2.01, 8.50)	2.68	0.227	0.49 (− 2.24, 3.22)	1.39	0.725	− 0.04 (− 2.29, 2.21)	1.15	0.975	2.44 (− 0.47, 5.34)	1.48	0.100	2.58 (− 2.66, 7.82)	2.67	0.334
Marital status (ref. widowed)	χ^2^ = 2.55 (*p* = 0.466)			χ^2^ = 3.17 (*p* = 0.366)			χ^2^ = 9.71 (*p* < 0.05)			χ^2^ = 6.76 (*p* = 0.080)			χ^2^ = 2.75 (*p* = 0.432)		
single	− 2.96 (− 8.74, 2.82)	2.95	0.315	− 1.42 (− 5.21, 2.37)	1.93	0.464	− 3.05 (− 5.73, − 0.36)	1.37	0.026	− 4.90 (− 8.72, − 1.08)	1.95	0.012	− 2.86 (− 8.91, 3.20)	3.09	0.355
married	− 1.84 (− 5.20, 1.52)	1.71	0.282	− 2.01 (− 4.61, 0.59)	1.33	0.130	0.36 (− 1.35, 2.07)	0.87	0.678	− 0.44 (− 2.62, 1.73)	1.11	0.689	1.53 (− 1.30, 4.37)	1.44	0.288
divorced	− 2.97 (− 8.93, 2.99)	3.04	0.328	− 1.94 (− 5.76, 1.89)	1.95	0.322	− 4.58 (− 8.63, − 0.54)	2.07	0.026	− 2.72 (− 6.57, 1.13)	1.96	0.166	− 2.06 (− 8.22, 4.10)	3.14	0.512
Cognitive functioning (*MMSE*^d^)	− 0.69 (− 1.44, 0.07)	0.38	0.075	0.68 (0.15, 1.21)	0.27	0.012	0.32 (− 0.09, 0.72)	0.21	0.125	0.16 (− 0.33, 0.65)	0.25	0.528	− 1.80 (− 2.58, − 1.03)	0.40	< 0.001

*95% CI* Confidence interval

^a^*WHOQOL*-*OLD* = *World Health Organization Quality of Life-OLD*-questionnaire [9, 10]

^b^*GDS-15* = *Geriatric Depression Scale* (short-form) [17–19]

^c^*A*ccording to the new *CASMIN* educational classification. Low = inadequately completed general education, general elementary education, basic vocational qualification or general elementary education and vocational qualification; Middle = intermediate vocational qualification or intermediate general qualification and vocational qualification, intermediate general qualification, general maturity certificate, vocational maturity certificate/general maturity certificate and vocational qualification; High = lower tertiary education-general diplomas/diplomas with vocational emphasis, higher tertiary education-lower level/higher level [23]

^d^*MMSE* *Mini-Mental-Status-Examination* [22]

## Discussion

The aim of the present study was to investigate the effect of depressive symptoms on overall QoL and its different facets in the oldest aged population from a longitudinal perspective. We found a strong effect of significant depressive symptoms on overall QoL as well as all the different facets of QoL according to the multidimensional measure of WHOQO-OLD for this age group.

These findings are in accordance with previous research for the entire old age range [for an overview, see 7, 24]. Numerous studies with a cross-sectional study design reported an association of significant depressive symptoms and QoL in the elderly population [25–28], however, with a cross-sectional design the direction of the effect remains uninvestigated. Sivertsen et al. [7] summarized 74 studies investigating the association of depression and QoL in old age in a systematic review and found 15 studies with a longitudinal study design also excluding participants with cognitive impairment and dementia as the present study. All of these studies investigated only global QoL and found, in concordance with our results, a significant effect of depressive symptoms at baseline on poorer QoL at follow-up. Only the longitudinal study of Enkvist et al. [11] of 681 community residents conducted in Sweden included individuals of the oldest age group, however, without information on the exclusion of cognitive impairment and dementia and investigating life satisfaction with one question. The results show a significant effect of depressive symptoms at baseline on lower life satisfaction 3 years later. Another methodical similar study of Ho et al. [29] in Singapore investigating 1844 community dwellers with a sample mean age of 66 years excluding people with dementia, reported that depression at baseline was negatively associated with health-related QoL for mental and physical components at baseline and follow-up. Ribeiro et al. [24] reported also an effect of depressive symptoms on overall QoL in the SHARE study for participants aged 50 and older. A comparison with the remaining longitudinal studies of Sivertsen et al. [7] is limited because of their different study design.

In addition, in each of the five facets of WHOQOL-OLD a significant effect of depressive symptoms was found, while the values of the different facets varied: the highest difference between participants with and without significant depressive symptoms was found in *social participation*, followed by the facets *activities*, *autonomy* and *sensory functions.* The lowest difference between both groups was found in facet *death and dying*. These results support the assumption that not all facets are affected in the same degree in the cause of depression [25]. Since until now no study with a methodical comparable study design including facets of QoL could be found, comparison with other studies is limited. Another (cross-sectional) representative German study with participants aged 60 years, assessing QoL also by the WHOQOL-OLD found a significant association of depressive symptoms only with the facets social participation, activities, as well as sensory abilities [28].

Depressive symptoms are often accompanied by self-esteem doubts, social withdrawal, and reduced motivation [30] that can lead to the feeling of low social participation, while the low difference in the facets *activities*, *autonomy,* and *sensory functions* can be explained by the expectations of ageing individuals, which changes are inherent in the aging process. The effect of depressive symptoms on QoL remained significant after adjustment for sociodemographic characteristics.

Consistent with the findings of the present study, Chachamovich et al. [25] also found the facet *death and dying* the lowest associated with depression in a younger sample (60 +) of mostly home-living people. Hussenoeder et al. [28] found no significant effect of depression on the facet *death and dying* in a cross-sectional design with individuals 60 years and older. Dragomirecká et al. [26] found the facet *death and dying* lowest associated with depressive symptoms in a sample of participants aged 60 years and older in a cross-sectional design.

We also found an effect of higher age on lower overall QoL score as well as on the facets *sensory abilities*, and *social participation*. Gobbens and Remmen [31] reported an association of older age with lower QoL in the four facets *sensory functions, autonomy, social participation, intimacy*, in a younger sample of Dutch people 50 years and older.

## Strengths and limitations

The present study is the first investigating the association of significant depressive symptoms and overall QoL as well as its different facets in the oldest population in Germany, with a longitudinal perspective. For investigation of QoL, a questionnaire especially developed for the elderly population—the WHOQOL-OLD—was used, including besides an overall QoL score different facets such as sensory abilities, social participation as well as death and dying which are especially relevant for the oldest population. However, the framework of the present study is accompanied by certain methodological limitations. First, the responses of the participants are subjective measures that may be subject to distortions. The distortion effects in the response behavior of the persons cannot be excluded with respect to the interview mode. Second, data on WHOQOL-OLD facet intimacy were not included and therefore study results cannot be compared with the overall QoL scores of other WHOQOL-OLD studies. Third, different cut-off scores for the GDS-15 were discussed. Although some studies used a stricter cutoff of 6 [26, 27], others set the score at 5 [32], or with 3–5 points for subclinical depressive symptoms [25]. Self-reported depressive symptoms using GDS-15 are not equivalent to clinically diagnosed depression. Future studies could examine clinical depression using standardized diagnostic instruments such as the structured clinical interview (SKID) [33]. Fourth, for the interpretation of the effects of depressive symptoms on QoL socio-demographic and health characteristics of incompleters and drop outs to FU8 and FU9 should be taken into account. On one hand, completers included in the analytic sample were younger and more often female had more depressive symptoms at baseline. On the other hand, drop outs at FU 8 and FU9 had a lower total QoL score as well as lower scores in some of the QoL facets (sensory abilities, activities, autonomy, and social participation, respectively) may have led to an underestimation of the effect of depressive symptoms on QoL. Fifth, detailed information on socio-economic state was not collected in the study, so only educational level could be included in the analyses, but for educational level neither a difference between the depressive and non-depressive subsample was shown, nor effects on overall QoL and its facets in the regression models. Furthermore, when interpreting the results, it should be taken into account, that the regression models were not adjusted for somatic comorbidity as well as use of medications, since available data did not have sufficient quality to be included.

## Conclusions/implications

With the present study, the association of significant depressive symptoms and the broad range of different facets of QoL were further enlightened in the population of the oldest age. Our findings have important implications for end-of-life health care considerations. In light of demographic changes and associated increased care needs in the old age population, the results underline the relevance of customized and targeted care planning and the development of customized interventions. To enable healthy and dignified aging, there is a need for socially and politically responsible and cost-effective approaches to support older people with depressive symptoms. Optimally, such interventions are primary preventative; but may also be included in secondary or tertiary prevention endeavors.

## Data Availability

The data that support the findings of this study are available on request from the corresponding author. The data are not publicly available due to privacy or ethical restrictions.

## Appendix

See Table 4

**Table 4:** Socio-demographic and health characteristics of the total analytical sample of GP patients at baseline compared to drop outs at FU8 and FU9 (*n* = 580)

	FU8					FU8/FU9					
Variables	Total (*n* = 580)	Drop-Out (*n* = 62; 10.7%)		Participants (*n* = 518; 89.3%)	*p*-value	Total (*n* = 580)	Drop-out (*n* = 121; 20.9%)		Participants (*n* = 459; 79.1%)		*p*-value
Age											
Range	85–99					85–99					
* M (SD)*	88.6 (2.7)		88.9 (2.8)	88.6 (2.7)	.304	88.6 (2.7)	88.8 (2.8)		88.5 (2.7)		0.321
Sex (*n*/%)										< 0.05	
Female	380 (65.5)		34 (54.8)	346 (66.8)	0.061	380 (65.5)		70 (57.9)	310 (67.5)		
Education (*n*/%)^a^										0.840	
Low	303 (52.2)		31 (50.0)	272 (52.5)		303 (52.2)		61 (50.4)	242 (52.7)		
Middle	179 (30.9)		20 (32.3)	159 (30.7)		179 (30.9)		40 (33.1)	139 (30.3)		
High	98 (16.9)		11 (17.7)	87 (16.8)	.932	98 (16.9)		20 (16.5)	78 (17.0)		
Marital status (*n*/%)										0.363	
Single	36 (6.2)		5 (8.1)	31 (6.0)		36 (6.2)		7 (5.8)	29 (6.3)		
Married	157 (27.1)		17 (27.4)	140 (27.0)		157 (27.1)		36 (29.8)	121 (26.4)		
Divorced	28 (4.8)		6 (9.7)	22 (4.3)		28 (4.8)		9 (7.4)	19 (4.1)		
Widowed	359 (61.9)		34 (54.8)	325 (62.7)	0.228	359 (61.9)		69 (57.0)	290 (63.2)		
Living situation (n/%)										0.482	
Private home	536 (92.4)		56 (90.3)	480 (92.7)		536 (92.4)		110 (90.9)	426 (92.8)		
Institution	44 (7.6)		6 (9.7)	38 (7.3)	0.511	44 (7.6)		11 (9.1)	33 (7.2)		
*MMSE*^b^											
Range	24–30					24–30					
* M (SD)*	28.0 (1.6)		27.7 (1.7)	28.1 (1.6)	0.077	28.0 (1.6)		27.7 (1.6)	28.1 (1.6)	< 0.05	
*GDS-15*^c^											
range	0–14					0–14					
* M (SD)*	2.6 (2.5)		3.7 (2.6)	2.4 (2.5)	< .001	2.6 (2.5)		3.3 (2.6)	2.4 (2.5)	< 0.001	
*WHOQOL-OLD*^d^											
Range	19–80					19–80					
* M (SD)*	55.3 (9.6)		51.7 (8.6)	55.7 (9.7)	< .001	55.3 (9.6)		52.7 (9.4)	55.9 (9.6)	< 0.001	
Sensory abilities	55.6 (17.1)		51.0 (17.6)	56.1 (17.0)	< 0.05	55.6 (17.1)		52.4 (18.3)	56.4 (16.7)	< 0.05	
Autonomy	53.1 (13.8)		49.9 (12.4)	53.5 (13.9)	0.052	53.1 (13.8)		50.6 (12.2)	53.8 (14.1)	< 0.05	
Activities	55.0 (10.9)		52.0 (11.1)	55.3 (10.9)	< 0.05	55.0 (10.9)		53.6 (11.2)	55.3 (10.9)	0.122	
Social participation	53.3 (13.7)		44.5 (15.5)	54.3 (13.1)	< 0.001	53.3 (13.7)		48.1 (14.4)	54.6 (13.2)	< 0.001	
Death and dying	59.4 (18.2)		60.8 (17.6)	59.2 (18.3)	0.507	59.4 (18.2)		58.8 (18.6)	59.5 (18.1)	0.688	

*n* frequencies, % percent, *M* Mean, SD Standard deviation

^a^*a*ccording to the new CASMIN educational classification. Low = inadequately completed general education, general elementary education, basic vocational qualification or general elementary education and vocational qualification; Middle = intermediate vocational qualification or intermediate general qualification and vocational qualification, intermediate general qualification, general maturity certificate, vocational maturity certificate/general maturity certificate and vocational qualification; High = lower tertiary education-general diplomas/diplomas with vocational emphasis, higher tertiary education-lower level/higher level [23]

^b^*MMSE* = *Mini-Mental-Status-Examination* [22]

^c^*GDS-15* = *Geriatric Depression Scale* (short-form) [17–19]

^d^*WHOQOL-OLD* = *World Health Organization Quality of Life-OLD*-questionnaire [9, 10]
